# Patient-centric assessment of rheumatoid arthritis using a smartwatch and bespoke mobile app in a clinical setting

**DOI:** 10.1038/s41598-023-45387-7

**Published:** 2023-10-25

**Authors:** Valentin Hamy, Christopher Llop, Christopher W. Yee, Luis Garcia-Gancedo, Aoife Maxwell, Wen Hung Chen, Ryan Tomlinson, Priyanka Bobbili, Julien Bendelac, Jessica Landry, Maral DerSarkissian, Mihran Yenikomshian, Elinor A. Mody, Mei Sheng Duh, Rachel Williams

**Affiliations:** 1 Value Evidence and Outcomes, GSK, Brentford, TW8 9GS UK; 2 Analysis Group Inc., Boston, USA; 3 Value Evidence and Outcomes, GSK, Philadelphia, USA; 4 Medicine Development Leaders, GSK, Philadelphia, USA; 5 Rheumatology Department, Reliant Medical Group, Auburn, USA

**Keywords:** Rheumatoid arthritis, Biomarkers, Health care

## Abstract

Rheumatoid arthritis (RA) is a fluctuating progressive disease requiring frequent symptom assessment for appropriate management. Continuous tracking using digital technologies may provide greater insights of a patient’s experience. This prospective study assessed the feasibility, reliability, and clinical utility of using novel digital technologies to remotely monitor participants with RA. Participants with moderate to severe RA and non-RA controls were monitored continuously for 14 days using an iPhone with an integrated bespoke application and an Apple Watch. Participants completed patient-reported outcome measures and objective guided tests designed to assess disease-related impact on physical function. The study was completed by 28 participants with RA, 28 matched controls, and 2 unmatched controls. Completion rates for all assessments were > 97% and were reproducible over time. Several guided tests distinguished between RA and control cohorts (e.g., mean lie-to-stand time [seconds]: RA: 4.77, control: 3.25; *P* < 0.001). Participants with RA reporting greater stiffness, pain, and fatigue had worse guided test performances (e.g., wrist movement [*P* < 0.001] and sit-to-stand transition time [*P* = 0.009]) compared with those reporting lower stiffness, pain, and fatigue. This study demonstrates that digital technologies can be used in a well-controlled, remote clinical setting to assess the daily impact of RA.

## Introduction

Rheumatoid arthritis (RA) is a chronic, systemic, and progressive autoimmune disease that affects an estimated 2 million Americans and 20 million people worldwide^1^. RA manifests in a range of symptoms that can fluctuate daily, including joint pain, stiffness, swelling, and fatigue^2–4^. Due to the progressive and fluctuating nature of the disease, frequent symptom assessment is paramount for a more accurate representation of the disease^5,6^.

RA symptoms are conventionally assessed using validated patient-reported outcome (PRO) measures, such as the health assessment questionnaire (HAQ), patient global assessment (PGA), visual analog scale (VAS), pain VAS, and duration of morning stiffness^7^. While well-developed PRO measures capture useful information regarding the symptoms, function, and impact of disease as experienced by patients^8^, they are subjective and provide only a snapshot of the patients’ lives, particularly if administered infrequently and inconsistently^4^. To provide a more complete picture of the patient’s status, there is a need for novel approaches to enable more frequent and sensitive monitoring of the patients’ function, disease impact, and quality of life in an objective manner to complement PRO data in clinical trials and clinical practice^9^.

Digital technologies offer the opportunity of continuously tracking a patient’s experience with RA and may allow for the collection of patient-centric assessments of disease activity in real-world settings. Furthermore, the use of familiar user-friendly consumer devices likely improves participant use throughout the study period^10^. The PARADE study demonstrated the feasibility of remotely collecting data on participants with RA in a real-world setting using a smartphone application, including PRO and objective sensor data on physical function assessment^5,11^. However, the PARADE study lacked clinical supervision and found that participant compliance decreased substantially over time. Another study used a wrist-worn sensor to assess morning activity patterns in participants with RA in a clinical setting and successfully recorded significant differences compared with healthy controls^12^.

Digital technologies have also been used to remotely monitor patients with other conditions, such as neurodegenerative diseases. A clinical trial measured daily activity in patients with Parkinson’s disease using a smartphone device with a pre-installed bespoke application and found significant differences in everyday motor behaviour compared with healthy controls^13^. In a Phase II trial, the same group demonstrated promising adherence, reliability, and validity of using a smartphone and smartwatch with a bespoke mobile application to collect data on participants’ performance of daily tasks^14^. An exploratory trial assessed physical functioning in patients with amyotrophic lateral sclerosis using an accelerometer, wireless hub, and microphone, which showed deterioration over time and moderate-to-strong correlations with the Revised Amyotrophic Lateral Sclerosis Rating Scale (ALSFRS-R), the gold standard measure of functional decline^15,16^. Daily physical functioning has also been assessed in patients with multiple sclerosis; sensor data provided the ability to distinguish from healthy controls as well as between disease severity groups^17^. These and other studies have set forth the use of digital health technologies in clinical research. European and US regulators have produced draft guidance for their use to collect data remotely in clinical trials and recommend ensuring the technology is fit-for-purpose through evidence-based feasibility and validation prior to clinical assessment^10,18–20^. This includes evaluation of established and/or novel endpoints, ensuring that the latter are clinically meaningful and capable of detecting aspects of disease progression that are important for patients^10^.

The main aim of this study was to investigate whether digital technologies are capable of remotely monitoring the daily impact of RA symptoms on physical functions associated with day-to-day activities in the home environment. To accomplish this, a series of objective tests were employed along with standard PROs in both RA and control cohorts, all measured using a smartphone with a bespoke mobile app. In auxiliary, the feasibility, reliability, and reproducibility of data capture using these digital technologies were assessed.

## Results

### Participant population

Of a total of 60 recruited participants, two participants with RA withdrew prior to data collection, while 28 participants with RA, 28 matched non-RA controls (referred to as “controls” hereafter), and 2 unmatched controls completed the study. RA and control cohorts had a mean (standard deviation [SD]) age of 58.8 (9.3) and 58.5 (9.9), respectively, and were mostly female (both 89%) and mostly white (86% vs 89%, respectively; Table 1 and Supplementary Table S1). Mean (SD) body mass index (BMI) was significantly higher in RA (31.4 [7.3]) versus control cohorts (25.8 [4.6], *P* < 0.001). There were 13/28 participants (46%) with moderate RA and 15/28 (54%) with severe RA. The majority of participants with moderate RA (85%) had ≥ 10 years since RA diagnosis, whereas participants with severe RA were mostly diagnosed 5–9 years prior (47%, *P* < 0.01; Table 2).

**Table 1:** Participant demographics.

Demographics	Controls (N = 28)	RA (N = 28)	*P*-value: RA vs controls*	Moderate RA^†^ (N = 13)	Severe RA^‡^ (N = 15)	*P*-value: severe vs moderate
Age, years, mean (SD)	58.5 (9.9)	58.8 (9.3)	0.36	56.9 (11.4)	60.4 (7.1)	0.33
Female, n (%)	25 (89)	25 (89)	1.00	11 (85)	14 (93)	0.92
BMI, mean (SD)	25.8 (4.6)	31.4 (7.3)	**0.001**	31.1 (5.9)	31.7 (8.6)	0.87
Ethnicity/race,^§^ n (%)			0.07			0.51
White	25 (89)	24 (86)		11 (85)	13 (87)	
Asian	3 (11)	0 (0)		0 (0)	0 (0)	
Black	0 (0)	1 (4)		0 (0)	1 (7)	
Other	0 (0)	3 (11)		2 (15)	1 (7)	
Hispanic, Latino/a, Spanish origin	0 (0)	5 (18)	0.09	2 (15)	3 (20)	0.82

Mean and SD were calculated using descriptive statistics. For continuous measures, a Wilcoxon signed-rank test was used to compare the collection rates of matched participants with RA and controls, and participants with severe vs moderate RA. For categorical measures, a Chi-square test of independence was used to compare the collection rates between RA vs control cohorts, and participants with severe vs moderate RA. *P*-values < 0.05 were considered to be statistically significant and are marked in bold. Due to rounding, n totals may not sum 100%

*Two participants with RA withdrew from the study before contributing data; therefore, to preserve matching groups for statistical tests, the matched controls were not included in these analyses.

^†^RAPID-3 score ≤ 12.

^‡^RAPID-3 score > 12.

^§^The “Hispanic, Latino/a, Spanish origin” ethnicity item was separate from the race items, so that people of that ethnicity may identify as being of a particular race (e.g., Black Latina); therefore, percentages in this category may add up to > 100%.

*BMI* body mass index, *RA* rheumatoid arthritis, *RAPID-3* routine assessment of patient index data 3, *SD* standard deviation.

**Table 2:** Clinical characteristics of participants with RA.

Clinical characteristics	RA (N = 28)	Moderate RA* (N = 13)	Severe RA^†^ (N = 15)	*P*-value: severe vs moderate
Years since RA diagnosis, n (%)				
< 2	2 (7)	0 (0)	2 (13)	**0.01**
2–4	3 (11)	2 (15)	1 (7)	
5–9	7 (25)	0 (0)	7 (47)	
≥ 10	16 (57)	11 (85)	5 (33)	
Current medication use for RA				
Any medication use, n (%)	28 (100)	13 (100)	15 (100)	0.93
Acetaminophen	10 (35)	4 (30)	6 (40)	
Anti-inflammatory pain killers	18 (64)	8 (61)	10 (66)	
Etanercept	6 (21)	4 (30)	2 (13)	
Methotrexate	15 (53)	7 (53)	8 (53)	
Corticosteroids	11 (39)	5 (38)	6 (40)	
Hydroxychloroquine	2 (7)	N/A	2 (13)	
Leflunomide	4 (14)	2 (15)	2 (13)	
Abatacept	3 (10)	1 (7)	2 (13)	
Adalimumab	3 (10)	1 (7)	2 (13)	
Infliximab	2 (7)	N/A	2 (13)	
Notable RA symptom, n (%)				
Joint pain	27 (96)	12 (92)	15 (100)	0.78
Fatigue	19 (68)	9 (69)	10 (67)	
Poor sleep	15 (54)	5 (38)	10(67)	
Mood variations	3 (11)	2 (15)	1 (7)	
Morning stiffness	15 (54)	6 (46)	9 (60)	
Walk and balance	11 (39)	3 (23)	8 (53)	
Other	0 (0)	0 (0)	0 (0)	

A Chi-square test of independence was used to compare participants with severe vs moderate RA. *P*-values < 0.05 were considered statistically significant and are marked in bold. Due to rounding, n totals may not sum 100%

*RAPID-3 score ≤ 12.

^†^RAPID-3 score > 12.

*N/A* not available, *RA* rheumatoid arthritis, *RAPID-3* routine assessment of patient index data 3.

### Construct validity: correlations between guided test performance and PRO measures

**Figure 1:**
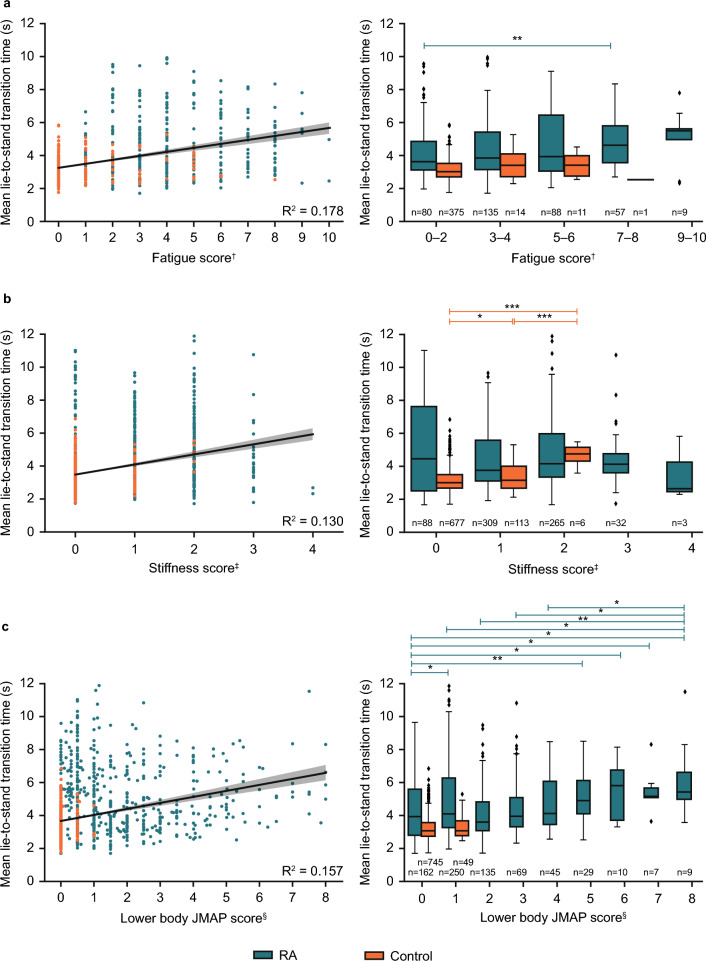
Correlations between lie-to-stand time and fatigue (**a**), stiffness (**b**), and lower body JMAP score (**c**). R^2^ values calculated using Spearman’s rank correlation. One-way ANOVA was used to compare differences in guided test performances and PRO measures. One-way ANOVA post hoc analyses were used to compare differences in guided tests between groups based on symptom severity as determined by PRO measures (*P < 0.05, **P < 0.01, ***P < 0.001). ^†^Fatigue was assessed through a single-item question asking participants to rate their worst level of fatigue over the past 24 h on a 10-point scale, ranging from “no fatigue” (0) to “as bad as you can imagine” (10). FACIT-Fatigue questionnaire was administered only on Days 1, 7, and 14, hence the fewer data points compared to stiffness and lower body JMAP score plots; ^‡^stiffness was assessed using a single-item question on the severity of morning stiffness experienced by the participant on a given day, with 5 response options provided, ranging from no stiffness (0) to very severe (4); ^§^lower body JMAP score was calculated by summing the scores for each hip, knee, and ankle joint (left and right; total = 6), plus the average score of all foot joints (10 joints per foot) and dividing by 2. Score could range from a minimum of 0 to a maximum of 12 (maximum reached in study was 8). *ANOVA* analysis of variance, *FACIT-Fatigue* functional assessment of chronic illness therapy—fatigue, *JMAP* joint-pain map, *PRO* patient-reported outcome, *RA* rheumatoid arthritis, *s* seconds.

Correlation analysis showed that higher fatigue, stiffness, and lower body joint-pain map (JMAP) scores correlated with longer mean lie-to-stand transition times (Fig. 1a–c), with fatigue showing the strongest correlation with the lie-to-stand test (correlation coefficient: 0.42). One-way analysis of variance (ANOVA) showed differences between guided test performance across levels of symptom severity, as assessed by PRO measures. This included lie-to-stand transition times and fatigue, stiffness, and the aggregated lower body JMAP metric (all *P* < 0.0001; Fig. 1a–c), dominant wrist angular velocity and dominant wrist pain (*P* < 0.001; Supplementary Fig. S1a), median wrist range of motion (ROM) and degree of stiffness (*P* < 0.001; Supplementary Fig. S1b), and sit-to-stand transition times and degrees of stiffness (*P* < 0.0001; Supplementary Fig. S1c).

Additional post hoc analyses showed that participants with RA and fatigue PRO scores of 0–2 performed the lie-to-stand test faster than those with fatigue of 7–8 (*P* = 0.046; Fig. 1a), and those with less severe lower body JMAP scores also had quicker times than those with higher JMAP scores (Fig. 1c). Participants with RA with a JMAP score of 0 had faster wrist movements than those with higher JMAP scores, whereas no significant differences were found with controls (Supplementary Fig. S1a). Participants with RA with no stiffness had greater range of wrist movement (*P* < 0.001; Supplementary Fig. S1b) and faster sit-to-stand transition times than those with a stiffness score of 2 (*P* = 0.009; Supplementary Fig. S1c).

### Clinical utility: comparisons of guided test and PRO scores between RA and control cohorts

Participants with RA had a greater maximum wrist ROM than controls (mean [SD] of dominant hand 165.1 [8.6] vs 156.9 [9.9] degrees; *P* < 0.001) but lower maximum velocity (mean [SD] of dominant hand 410.5 [163.5] vs 509.1 [134.1] deg/s [s]; *P* = 0.03; Table 3). Participants with RA had longer transition times for the sit-to-stand (mean [SD] 1.74 s [0.52] vs 1.49 s [0.25]; *P* = 0.03) and lie-to-stand tests (mean [SD] 4.77 s [1.70] vs 3.25 s [0.61]; *P* = 0.001) vs controls, but not for the stand-to-sit transition. No notable differences were found between participants with moderate and severe RA for wrist ROM, sit-to-stand, and lie-to-stand tests, although the transition time for the lie-to-stand test was longer in severe versus moderate RA cohorts (mean [SD] 4.90 s [1.65] vs 4.62 s [1.82]; *P* = 0.59).

**Table 3:** Guided test metrics.

	Controls	RA	*P*-value: RA vs controls*	Moderate RA^†^	Severe RA^‡^	*P*-value: severe vs moderate
	(N = 27)	(N = 27)		(N = 13)	(N = 15)	
Wrist ROM test^§^,**						
Both hands, mean (SD)						
Median ROM, deg	145.1 (10.5)	153.3 (8.8)	** < 0.001**	154.1 (9.0)	153.5 (9.2)	0.84
Max ROM, deg	158.6 (8.7)	165.5 (8.4)	**0.002**	167.8 (8.2)	164.1 (8.6)	0.28
Median velocity, deg/s	152.2 (60.4)	106.6 (41.2)	**0.001**	118.1 (51.0)	91.0 (32.2)	0.16
Max velocity, deg/s	516.3 (134.0)	417.3 (164.0)	**0.03**	454.1 (160.3)	371.7 (165.6)	0.17
Dominant hand, mean (SD)						
Median ROM, deg	145.5 (10.3)	154.2 (9.2)	** < 0.001**	155.2 (10.6)	154.4 (8.7)	0.84
Max ROM, deg	156.9 (9.9)	165.1 (8.6)	** < 0.001**	167.9 (8.3)	163.5 (8.9)	0.21
Median velocity, deg/s	155.8 (63.0)	108.4 (44.2)	** < 0.001**	122.1 (55.8)	90.2 (32.6)	0.08
Max velocity, deg/s	509.1 (134.1)	410.5 (163.5)	**0.03**	446.8 (159.6)	364.2 (166.8)	0.16
Non-dominant hand, mean (SD)						
Median ROM, deg	144.7 (11.2)	152.4 (9.4)	**0.005**	153.0 (8.6)	152.6 (10.4)	0.91
Max ROM, deg	160.3 (8.1)	165.9 (8.7)	**0.005**	167.8 (8.2)	164.8 (9.1)	0.50
Median velocity, deg/s	148.6 (59.4)	104.7 (42.4)	**0.002**	114.1 (50.1)	91.8 (36.2)	0.17
Max velocity, deg/s	523.6 (138.4)	424.0 (170.3)	**0.02**	461.3 (166.2)	379.3 (170.9)	0.15
Sit-to-stand and lie-to-stand tests^††^						
Transition times, mean (SD)						
Sit-to-stand, s	1.49 (0.25)	1.74 (0.52)	**0.03**	1.74 (0.61)	1.74 (0.44)	0.66
Stand-to-sit, s	1.75 (0.30)	1.95 (0.56)	0.16	1.98 (0.64)	1.92 (0.49)	0.70
Lie-to-stand, s	3.25 (0.61)	4.77 (1.70)	**0.001**	4.62 (1.82)	4.90 (1.65)	0.59
Stand-to-lie, s	3.17 (0.54)	4.24 (1.27)	**0.002**	4.02 (1.27)	4.45 (1.28)	0.41

Wilcoxon signed-rank test was used to compare the collection rates of matched RA vs control cohorts, and participants with severe vs moderate RA. *P*-values < 0.05 were considered to be statistically significant and are marked in bold.

*Two participants with RA withdrew from the study before contributing data; therefore, to preserve matching groups for statistical tests, the matched controls were not included in these analyses.

^†^RAPID-3 score ≤ 12.

^‡^RAPID-3 score > 12.

^§^One control was excluded due to data quality issues; their matching participant with RA was excluded from the main RA column but included in the Moderate RA column.

**98.3% of completed wrist exercises passed the QC process and were included in the analysis.

^††^95.4% of completed lie-to-stand exercises and 84.1% of completed sit-to-stand exercises passed the QC process and were included in the analysis.

*Deg* degrees, *deg/s* degrees per second, *max* maximum, *QC* quality control, *RA* rheumatoid arthritis, *RAPID-3* routine assessment of patient index data 3, *ROM* range of motion, *s* seconds, *SD* standard deviation.

The gait and peg tests reported similar results between RA and control cohorts, determining these as less useful measures for testing physical functioning of participants with RA.

Participants with RA had significantly worse PRO scores than controls (Supplementary Table S2). In addition, patients with severe RA had worse scores for most PROs compared with the moderate RA group, reaching statistical significance for HAQ Disability Index (HAQ-DI), RA Symptom and Impact Questionnaire (RASIQ) pain, RASIQ stiffness, morning and afternoon stiffness, pain, PGA, and Short Form 36 (SF-36) physical component summary (PCS) scores. The results showed that, in almost all joints, participants with RA had significantly higher average pain scores over the duration of the study than controls (*P* ≤ 0.004), whereas there was no significant difference in pain scores in any joint between participants with moderate versus severe RA.

### Feasibility: task completion and compliance rates

Completion rates were high in both cohorts, with mean completion rates for both PROs and guided tests of 97% for the RA cohort and 99% for the control cohort. Similarly, the quality control (QC) process showed a high rate of correct exercise completion: 98% for the wrist test, 99.9% for walk exercises, 84% for completed sit-to-stand tests, and 95% for completed lie-to-stand tests.

On all study days, the majority of participants in RA and control cohorts wore the Apple Watch for > 85% (RA: 82%; control: 90%) and many even for > 90% (RA: 64%; control: 50%) of the day, which was inclusive of charging time (approximately 2 h per day). Nearly all participants in RA and control cohorts had 100% completion of assigned daily tasks on all 14 study days. Mixed effects linear models found no significant effect of study day on wear rates or task completion rates.

### Reproducibility and reliability: effect over time of guided test performance and inter-rater reliability analysis for RA and controls

A univariate mixed effects linear model found no significant changes over time in any of the PRO measures or in the lie-to-stand transition times throughout the 14-day study period (*P* < 0.113; Supplementary Fig. S2a); however, there was an increase in wrist angular velocity (*P* < 0.001, Supplementary Fig. S2b) and a decrease in the sit-to-stand transition time (*P* < 0.001; Supplementary Fig. S2c) and maximum ROM (*P* = 0.043) between Day 1 and Day 14. There was also a significant decrease in maximum ROM in controls (*P* = 0.030) but not RA participants (*P* = 0.750) over the study period.

Intra-class coefficients (ICCs) for controls ranged from 0.599 to 0.852, while those for participants with RA ranged from 0.722 to 0.897, with the highest ICCs generally for the wrist and standing (vs sitting/lying) exercises for both RA and control cohorts.

## Discussion

Although digital endpoints based on guided tests administered through a smartphone app have been explored in other diseases^13,14,17^, the application to RA is relatively unexplored. To our knowledge, PARADE was the first of these studies in RA with a subsequent study that used sit-to-stand tasks but in a relatively small study population size (N = 45)^5,21^. The current study adds to the limited available evidence and demonstrated moderate correlations between guided test-derived measures and PRO measures, which were indicative of construct validity, while RA and control cohorts were distinguishable by their guided test scores, providing evidence of clinical utility. Also, this study verified the feasibility of using digital technologies to monitor the physical function and impact of disease on participants with RA remotely over a 14-day period in a controlled clinical setting. Compliance rates were exceptionally high, and results were generally reproducible over time.

Correlations of the lie-to-stand test results with PRO scores (fatigue, stiffness, lower body JMAP metric) demonstrate how digital measures can be linked to different symptoms while providing objectivity to conventional assessments. In particular, the lie-to-stand test relates closely to the action of getting out of bed and is therefore a valuable metric, as the ability to perform daily activities is a key indicator of patients’ quality of life in RA^2^. The PRO scores in this study that were indicative of greater pain or discomfort were generally associated with worse guided test scores in the RA cohort. Specifically, those with a dominant wrist pain score of 3 had a reduced median angular velocity of their dominant wrist than those with a pain score of 1. Similarly, those with higher body JMAP scores of 6–8 had slower lie-to-stand transition times than those with JMAP scores of 0–4. In summary, this represents the initial stages of validation for novel digital endpoints in RA, with assessment of clinical validity in the context of an interventional clinical trial being the logical next stage of progression.

Regarding clinical utility, the guided tests separated RA and control cohorts, as did standard PRO scores; however, results should be treated with caution considering the descriptive nature of the statistical analyses used in this study. Longer sit-to-stand (RA: 1.74 s; control: 1.49 s; *P* = *0.03*) and lie-to-stand times (RA: 4.77 s; control: 3.25 s; *P* = *0.001*) in RA participants compared with controls are likely a result of their symptoms (fatigue, stiffness, pain) directly impacting their performance speeds of these actions. Furthermore, symptoms commonly fluctuate in RA^3^, which could have contributed to the greater variability of guided test performance among the RA cohort compared with controls. Although guided tests were unable to differentiate between moderate and severe RA cohorts in this study, most PRO measures (pain, stiffness, fatigue, and lower body JMAP) were able to separate moderate from severe RA. Wrist angular velocity was inversely related to increasing levels of wrist pain (dominant wrist pain 0 vs 1–3; *P* < 0.05), consistent with the PARADE study findings^5^. In a study using smartphones in a clinical trial setting, worse activity-based scores were shown for participants with Parkinson’s disease compared with healthy controls, and activity tests were related to symptom severity. Furthermore, guided active tests identified abnormalities that were missed in patient examinations, suggesting that digital assessments may be more sensitive and may provide additional insights compared with conventional measures^13^.

Differences were found between the performance over time of the individual guided tests. For example, the lie-to-stand test results were consistent over the duration of the study period while the wrist angular velocity and sit-to-stand tests saw improvements in scores over time. Given that the latter two tests were simple one-step motions, patients are likely to have learned and become more confident with the tests, leading to quicker motions and shorter transition times, as the study continued. Similarly, the decrease in wrist ROM over time in controls compared with stable metrics shown in the RA cohort may be due to increased awareness of the assessment’s objective by the latter.

Passive data collection and task completion rates were very high across all participants over the study period. These rates were higher than those in the PARADE study in which, by Week 2 of 12, only 40.6% of participants had provided data, despite 73.2% of participants at study initiation showing preference for participating in a study using a mobile app over one conducted in a clinic^5^. Potential reasons for the higher rates of retention in this study include involving clinician-recruited participants with upcoming visits as opposed to self-recruitment, confirming clinical diagnosis of the participants with RA, using a robust monitoring system to encourage task completion, compensating participants, use of consumer technology that many participants would already be familiar with, and the relatively short study duration. Furthermore, involving an advisory board of participants with RA in the design of the study and mobile application and providing patient training for performing the various study assessments may have contributed to enhanced participant engagement in this study^22^. The participant responses to the beta test fed into optimizing the study design and technology to proactively tackle any common user issues. Reassuringly, participants responded with “Strongly agree” (77%) or “Agree” (21%) after the study when asked if they would be involved in an identical future study. Similarly, the Remote Monitoring of RA (REMORA) study also incorporated patient feedback to help optimize development of their mobile app and found strong patient engagement over the 3-month study period^23^. Lastly, the current study included more robust quality check systems than the PARADE study^11^ to ensure that only data from correctly performed tests were analyzed. Together, this highlights the importance of incorporating appropriate training, involving patients in device/application development, and including data integrity checks in studies involving remote monitoring and digital technologies.

While this study had encouraging results with its various objectives, there were some limitations to consider. To allow for one-to-one follow-up, a relatively small population was included over a short study period. Also, the study population lacked demographic variability due to the involvement of only one clinical site. Thus, findings should be interpreted within this context. It should be noted that 75% of the RA cohort had a college/graduate level education and therefore does not represent low education level populations, while engagement with the digital technologies tested relied upon a certain level of education/experience. However, by utilizing user-friendly and familiar devices, it is anticipated the study findings will be applicable across a wider patient demographic. As this was an exploratory study into the use of devices to measure patient function, we did not assess whether the measures conducted in this study would be sensitive to drug treatment effects or disease progression. Additionally, potential confounding factors such as the BMI difference between RA and control cohorts could have impacted the sit-to-stand or lie-to-stand metrics; however, associations between guided test performance and PRO measures were not altered when BMI was controlled for (data not shown). Finally, as this study was not adjusted for multiplicity or powered for hypothesis testing, statistical comparisons could be used only for quantification/descriptive purposes.

Future developments would include creating composite endpoints that combine both active and passive monitoring, as well as PRO and other clinical measures, potentially providing a more robust means to differentiate control and RA cohorts and disease severity. Future work in a study population with a wider demographic would involve a more targeted and reduced set of assessments (e.g., only including the lie-to-stand guided test, which showed the greatest promise in this study) for simpler use and implementation. Further longitudinal studies are warranted to evaluate the potential of using the technology and guided tests in a remote clinical trial setting. This will help substantiate the findings in larger populations with appropriate statistical power to enable hypothesis testing.

Few studies have used digital endpoints based on guided tests administered through smartphones to assess symptoms of RA^5,21^, emphasizing this as a relatively unexplored field of study. Although these findings require further validation with a larger group of participants over a longer study period, the guided tests and the digital technologies used showed capability of capturing consistent and reliable information on the physical functioning of participants, thus offering a more holistic picture of a patient’s disease alongside conventional measurements. Participant retention and compliance were high, with consistent engagement with the mobile app throughout the course of the study. Ultimately, digital technologies have the potential to gain greater insights into the impact of patient symptoms and their ability to perform daily tasks. This will allow for a more enhanced and patient-centric understanding of the effect of novel medicines, leading to improved efficacy assessment as well as faster decision-making in future trials.

## Methods

### Study design

**Figure 2:**
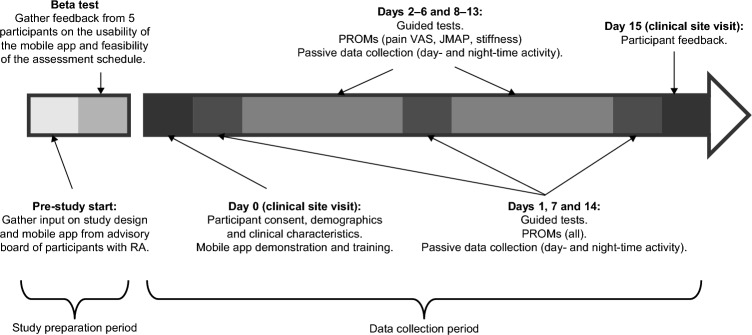
Study design. *JMAP* joint-pain map, *PROM* patient-reported outcome measure, *RA* rheumatoid arthritis, *VAS* visual analog scale.

This was a prospective study of participants with moderate to severe RA and 28 controls, matched on age (± 3 years), sex, and race. A sample size of 60 was chosen to facilitate one-to-one follow-ups. RA and control participants were recruited from Reliant Medical Group’s Rheumatology clinic and General Medical clinic (Worcester, Massachusetts). All participants were given an Apple Watch Series 4 and an iPhone 7 that had a preloaded, bespoke, study-specific mobile application. In-person training was provided by a site study coordinator on how to use the devices and application, perform the guided tests, and complete the PRO measures. Participants were required to complete PRO measures either weekly, daily, or twice a day, and guided tests twice a day for 14 days (Fig. 2; Supplementary Table S3). Data were transmitted in near real time to monitor compliance and for study coordinators to contact participants to encourage task completion if necessary.

Prior to the study initiation, an advisory board of patients with RA (not participating in the study) provided input on the study design and mobile app; this was followed by a beta test over a 5-day period involving 5 participants with RA to gather feedback on the usability of the app, clarity of instructions, and feasibility of the assessment schedule. Feedback from the beta test included difficulty in remembering to charge the devices, inconsistent syncing of data to the backend server, and limitations of the iPhone leg strap. In response to this feedback, the training was updated to include more specific instructions on when to charge the devices, the software was updated to improve data syncing, and the leg strap was redesigned along with providing a detailed pictorial guide for how to attach it.

### Study objectives

The following study objectives were investigated:

1. Construct validity: correlations between guided test performance and PRO measure severity.
2. Clinical utility: differences in guided test performance and PRO measure scores between RA and control cohorts.
3. Feasibility: completion rates of study assessments and Apple Watch wear rates in RA and control cohorts.
4. Repeatability and reproducibility: changes in guided test performance and PRO measures over time in RA and control cohorts.

### Ethics

All documentation, including the study protocol, any amendments, and informed consent procedures, was reviewed and approved by Reliant Medical Group’s Institutional Review Board. All participants provided written informed consent before any study procedures were undertaken. The study was conducted in accordance with the International Committee for Harmonisation principles of Good Clinical Practice and the Declaration of Helsinki.

### Participant selection

The full list of participant inclusion and exclusion criteria is included in Supplementary Table S4. Briefly, participants with RA were recruited from the Reliant Medical Group by clinicians during a clinical visit if they had a clinically verified diagnosis of moderate to severe RA, with severity assessed using Routine Assessment of Patient Index Data 3 (RAPID-3; score ≤ 12: moderate RA; score > 12: severe RA). Controls were outpatients from the Reliant Medical Group and were excluded if they had prior or current diagnosis of a rheumatological disorder, inflammatory disorder, malignancy, or other relevant diseases.

### Study assessments

The schedule of assessments and example screenshots of the bespoke mobile application used to collect the metrics from these assessments are provided in Supplementary Table S3 and Supplementary Fig. S3, respectively.

For the guided tests, the iPhone was used to collect accelerometer and gyroscope data as participants performed predefined guided physical functioning tests. The guided exercises were designed using clinical and patient feedback to test aspects of participants’ functionality that are likely impacted by the symptoms that matter most to patients with RA (i.e., joint pain, stiffness, fatigue, and sleep)^2^. Participants were instructed to perform each guided test daily, once in the morning (immediately after waking up) and once in the afternoon, to assess the change in stiffness throughout the day. The wrist ROM test is described in detail in the PARADE study^5^. Briefly, while holding the iPhone facing upwards over the edge of a table, participants flexed and extended their wrist joint to its maximum angle (without going beyond the zone of comfort), repeating the motion for 10 s. The test was done once in both hands. For the sit-to-stand test, participants sat on a chair with the iPhone strapped to their upper right thigh and with their arms crossed over their chest, then stood up and sat down 5 times at their own pace. Average times taken to transition from sitting to standing and from standing to sitting were extracted from accelerometer and gyroscope data. For the lie-to-stand test, participants lay on a bed with their legs stretched and the iPhone strapped to their upper right thigh, then stood up on the floor twice at their own pace. Average times taken to transition from lying to standing and from standing to lying were extracted from accelerometer and gyroscope data. Other guided tests included the gait test and the 9-hole peg test (ResearchKit; Apple Inc., CA, USA)^5^. In the gait test, participants were asked to attach the iPhone to their right thigh and walk in a straight line for 30 s. The 9-hole peg test, which measures hand dexterity, asks participants to use two fingers on their left hand to drag a round ‘peg’ on the iPhone screen into a ‘hole’ elsewhere on the screen and then use two fingers on their right hand to remove the peg from the hole. The Apple Watch was also used to continuously collect background accelerometer data to passively measure daily and night-time activity counts, the data of which are not reported in this article.

PROs were assessed on Days 1, 7, and 14, and included the following: Functional Assessment of Chronic Illness Therapy—Fatigue (FACIT-Fatigue) to assess fatigue^28^; HAQ-DI and SF-36^29^ questionnaires to indicate impacts on participant’s quality of life^24,30,31^; Patient-Reported Outcomes Measurement Information System (PROMIS) Pain Interference to assess how pain interferes with participants’ daily wellbeing and PROMIS Sleep Disturbance to assess sleep quality^32^; RASIQ to quantify symptom severity and its impact on the participant^4^. RA-specific assessments (RASIQ, PGA, stiffness) were not administered to controls.

Short questionnaires were administered every day, except for morning stiffness that was done on Days 2–6 and 8–13. Morning stiffness and severity of stiffness were assessed from responses to questions 11, 12, and 13 from RASIQ^4^. The JMAP recorded the number and severity of painful joints experienced at a given time from 55 prespecified joints presented on a body map; pain was scored as no pain, mild pain, moderate pain, or severe pain^11^. Pain VAS assessed pain severity on a scale ranging from 0 mm (no pain) to 100 mm (worst pain)^33^. PGA measured overall the way RA affects participants and/or disease activity using a single-item question and the VAS scoring^34^. A global assessment of fatigue over the previous 24 h was measured on a 10-point scale, ranging from “no fatigue” (0) to “as bad as you can imagine” (10).

### Guided test algorithms

Details of the algorithms for the wrist ROM test, sit-to-stand test, and lie-to-stand test are provided in the Supplementary methods S1. An illustrative flow chart of the algorithm for the lie-to-stand test is shown in Supplementary Fig. S4 and has previously been reported for the wrist ROM test^11^.

### Data quality assessment

Automated and manual data quality assessments were conducted throughout the study to ensure that the data analyzed were only from tests that had been performed correctly. The wrist test quality check was performed manually, and both manual and algorithmic quality checks were performed for the walk test, sit-to-stand test, and lie-to-stand test. Tests that were clearly performed incorrectly were removed from the sample.

### Statistical analyses

Descriptive statistics were used for demographics and clinical characteristics, PRO measures, and guided tests. Wilcoxon signed-rank tests were used for matched (i.e., participants with RA vs controls and morning vs afternoon) and rank sum tests for unmatched (i.e., participants with moderate vs severe RA) comparisons. Nonparametric tests were used, as normal distribution could not be assumed due to the small sample size. Trends in time were assessed using univariate mixed effects models, with study day as a fixed effect and individual differences as random effects. ICCs were calculated to measure the consistency of guided tests over time for each participant using a two-way mixed effect, single rater, consistency convention; a higher ICC indicated a more regular test performance over the study period than a lower ICC. Correlations between PROs and guided tests were assessed using Pearson correlation coefficients, and one-way ANOVA was conducted using the Kruskal–Wallis test by ranks, with Mann–Whitney U-tests for post hoc pairwise comparisons. There was no adjustment for multiplicity in this study, and the study was not powered for hypothesis testing; therefore, *P*-values were used for quantification/descriptive purposes only.

### Supplementary Information

Supplementary Information.

## Data Availability

Anonymised individual participant data that support the findings of this study are available from the corresponding author, upon reasonable request and subject to GSK’s approval.
