# Supplementary material for: Patient-centric assessment of rheumatoid arthritis using a smartwatch and bespoke mobile app in a clinical setting
Source: Sci Rep. 2023 Oct 25;13:18311. doi: 10.1038/s41598-023-45387-7 (PMC10600111; doi:10.1038/s41598-023-45387-7)
Supplement: Supplementary file 1 — Supplementary Information. [file 41598_2023_45387_MOESM1_ESM.pdf]

Patient-Centric Assessment of Rheumatoid Arthritis Using a Smartwatch and Bespoke Mobile App in a Clinical Setting

Valentin Hamy[1,*,†], Christopher Llop[2,†], Christopher W Yee[2], Luis Garcia-Gancedo[1], Aoife Maxwell[1], Wen Hung Chen[3], Ryan Tomlinson[4], Priyanka Bobbili[2], Julien Bendelac[2], Jessica Landry[2], Maral DerSarkissian[2], Mihran Yenikomshian[2], Elinor A Mody[5], Mei Sheng Duh[2,‡], Rachel Williams[3,‡]

[1]Value Evidence and Outcomes, GSK, UK; [2]Analysis Group Inc., USA; [3]Value Evidence and Outcomes, GSK, USA; [4]Medicine Development Leaders, GSK, USA; [5]Rheumatology Department, Reliant Medical Group, USA.

*Corresponding author; [†]Joint first authors; [‡]Joint last authors

Supplement

Supplementary methods. Guided test algorithms

For the wrist range-of-motion (ROM) test, the validation and accuracy of the algorithm has previously been reported [5,11]. Briefly, gravity vector information collected by the iPhone accelerometer was converted to angular positions. ROM was calculated from the difference between angular extrema. Angular velocity was calculated from rotation rate information collected by the iPhone gyroscope.

For the lie-to-stand test, the validated algorithm was based on a previously reported method that used wearable sensors [35], which was then further adapted for the sit-to-stand test. Briefly, the algorithms determined when participants were in a lying, sitting, or standing position using gravity vectors from the iPhone gyroscope data. The y-gravity and acceleration vectors were analyzed to determine sit-to-stand and lie-to-stand transitions, while rotation angles calculated from the x-, y-, and z-gravity vectors determined if participants completed valid transitions during the exercises.

Supplementary Table 1. Additional demographics and clinical characteristics

	Controls (N = 28)	RA (N = 28)	*P*-value: RA vs Controls *	Moderate RA† (N = 13)	Severe RA‡ (N = 15)	*P*-value: Severe vs Moderate
**Demographics**						
**Height, inches, mean (SD)**	65.2 (3.2)	64.5 (3.4)	0.18	64.6 (3.6)	64.4 (3.4)	0.96
**Education level, n (%)**			0.40			0.97
Middle school	0 (0)	0 (0)		0 (0)	0 (0)	
High school	4 (14)	7 (25)		3 (23)	4 (27)	
College	14 (50)	15 (54)		7 (54)	8 (53)	
Graduate school	10 (36)	6 (21)		3 (23)	3 (20)	
**Clinical characteristics**						
**Additional medical conditions§**						
Any additional conditions, n (%)	28 (100)	28 (100)	1.00	13 (100)	15 (100)	1.00
Number of conditions,** mean (SD)	1.1 (0.3)	1.4 (0.7)	0.07	1.5 (0.9)	1.3 (0.6)	0.84
**Smoking status, n (%)**						
Never	20 (71)	16 (57)	0.11	7 (54)	9 (60)	0.45
Previous	8 (29)	8 (29)		3 (23)	5 (33)	
Current	0 (0)	4 (14)		3 (23)	1 (7)	
**Number of current medications, mean (SD)††**	N/A	2.9 (1.1)	N/A	2.7 (1.2)	3.1 (1.0)	0.38
**Past medication use for management of RA**	N/A		N/A			0.57
Any medication use, n (%)		27 (96)		13 (100)	14 (93)	
Methotrexate		11 (41)		4 (31)	7 (50)	
Corticosteroids		11 (41)		6 (46)	5 (36)	
Acetaminophen		5 (19)		3 (23)	2 (14)	
Anti-inflammatory pain killers		12 (44)		5 (38)	7 (50)	
Opioids		2 (7)		N/A	2 (14)	
Gold sodium thiomalate		4 (15)		3 (23)	1 (7)	
Hydroxychloroquine		8 (30)		5 (38)	3 (21)	
**Number of past medications,§ mean (SD)**	N/A	3.6 (2.1)	N/A	3.5 (2.4)	3.7 (1.9)	0.84

For continuous measures, a Wilcoxon signed-rank test was used to compare the collection rates of matched participants with RA vs control participants, and participants with severe vs moderate RA. For categorical measures, a Chi-square test of independence was used to compare the collection rates between RA vs control cohorts, and participants with severe vs moderate RA. *P*-values <0.05 were considered statistically significant and are marked in bold. Due to rounding, n totals may not sum 100%.

*Two participants with RA withdrew from the study before contributing data; therefore, to preserve matching groups for statistical tests, the matched controls were not included in these analyses; †RAPID-3 score ≤12; ‡RAPID-3 score >12; §these conditions include asthma, chronic obstructive pulmonary disease, depression or anxiety, diabetes, fibromyalgia, heart problems, hypertension, osteoarthritis, and recent knee surgery (within past 6 months); **calculated among participants with ≥1 additional condition; ††calculated among participants with non-zero medication use.

N/A, not available (data not collected for controls); RA, rheumatoid arthritis; RAPID-3, Routine Assessment of Patient Index Data 3; SD, standard deviation.

Supplementary Table 2. Patient-reported outcome (PRO) test scores

**PRO Survey**	**Possible Score Range**	**Controls (N=28)**	**RA (N=28)**	***P*-value: RA vs Controls***	**Moderate RA† (N=13)**	**Severe RA‡ (N=15)**	***P*-value: Severe vs Moderate**
**Long-form questionnaires, mean (SD)§**							
FACIT-Fatigue**	0–52	49.4 (2.2)	35.5 (6.8)	**<0.001**	37.8 (4.7)	33.3 (7.9)	0.19
HAQ-DI	0.0–3.0	0.0 (0.0)	0.8 (0.4)	**<0.001**	0.7 (0.3)	1.0 (0.4)	**0.0057**
PROMIS pain††	39.8–81.2	41.8 (3.9)	56.8 (6.2)	**<0.001**	54.5 (7.1)	58.9 (4.5)	0.05
PROMIS sleep	29.7–78.0	49.2 (2.4)	52.0 (3.2)	**0.0063**	52.1 (3.4)	51.9 (3.2)	0.66
RASIQ pain	0 –100	2.2 (4.7)	43.6 (17.4)	**<0.001**	32.8 (16.4)	53.6 (11.3)	**<0.001**
RASIQ impact	0–100	45.1 (3.9)	50.7 (5.5)	**<0.001**	51.1 (4.9)	50.4 (6.2)	0.87
RASIQ stiffness	0–100	4.8 (8.1)	41.9 (16.9)	**<0.001**	33.5 (18.1)	49.6 (11.7)	**0.024**
SF-36‡‡							
**Raw score, mean ± SD [median]**							
Physical functioning	0–100	97.9 ± 4.1 [100.0]	53.1 ± 24.1 [52.5]	**<0.0001**	64.9 ± 20.2 [65.0]	43.0 ± 23.1 [33.3]	**0.0193**
Role-functioning, physical	0–100	97.5 ± 5.3 [100.0]	66.8 ± 20.9 [64.6]	**<0.0001**	75.4 ± 18.7 [81.2]	59.4 ± 20.4 [58.3]	**0.0477**
Body pain	0–100	91.5 ± 10.4 [94.7]	54.9 ± 17.2 [51.3]	**<0.0001**	64.7 ± 17.2 [64.0]	46.5 ± 12.4 [49.7]	**0.0117**
General health	0–100	84.9 ± 15.3 [88.7]	43.8 ± 20.5 [42.2]	**<0.0001**	48.3 ± 20.9 [49.0]	40.0 ± 20.0 [36.3]	0.4253
Vitality	0–100	81.3 ± 11.6 [80.2]	46.7 ± 17.9 [45.8]	**<0.0001**	49.0 ± 14.5 [46.9]	44.8 ± 20.7 [41.7]	0.4404
Social functioning	0–100	99.0 ± 3.5 [100.0]	77.8 ± 21.3 [79.2]	**0.0001**	82.1 ± 20.9 [87.5]	74.1 ± 21.7 [77.1]	0.3545
Role-functioning, emotional	0–100	97.0 ± 5.9 [100.0]	86.2 ± 18.5 [94.4]	**0.0141**	87.7 ± 19.6 [98.6]	84.9 ± 18.1 [91.7]	0.4715
Mental health	0–100	88.2 ± 7.5 [88.3]	71.7 ± 19.5 [75.8]	**0.0005**	74.0 ± 17.6 [80.0]	69.6 ± 21.4 [75.0]	0.5371
**Standardized score, mean ± SD [median]**							
Physical functioning	0–100	56.7 ± 1.6 [57.5]	39.6 ± 9.2 [39.4]	**<0.0001**	44.1 ± 7.7 [44.1]	35.7 ± 8.9 [32.0]	**0.0193**
Role-functioning, physical	0–100	56.3 ± 1.9 [57.2]	45.2 ± 7.5 [44.4]	**<0.0001**	48.3 ± 6.7 [50.4]	42.6 ± 7.3 [42.2]	**0.0449**
Body pain	0–100	58.6 ± 4.2 [59.9]	43.8 ± 6.9 [42.4]	**<0.0001**	47.8 ± 6.9 [47.5]	40.4 ± 5.0 [41.7]	**0.0117**
General health	0–100	59.3 ± 7.3 [61.1]	39.8 ± 9.7 [39.0]	**<0.0001**	41.9 ± 10.0 [42.3]	38.0 ± 9.5 [36.2]	0.4253
**Physical component summary**	0–100	57.7 ± 3.6 [58.8]	39.4 ± 8.2 [38.6]	**<0.0001**	43.8 ± 7.4 [44.0]	35.6 ± 7.0 [35.6]	**0.0101**
Vitality	0–100	61.5 ± 5.5 [61.0]	45.1 ± 8.5 [44.7]	**<0.0001**	46.2 ± 6.9 [45.2]	44.2 ± 9.9 [42.7]	0.4404
Social functioning	0–100	56.9 ± 1.4 [57.3]	48.4 ± 8.5 [49.0]	**<0.0001**	50.2 ± 8.4 [52.3]	47.0 ± 8.7 [48.1]	0.3681
Role-functioning, emotional	0–100	54.9 ± 2.5 [56.2]	50.4 ± 7.7 [53.8]	**0.0165**	51.0 ± 8.2 [55.6]	49.9 ± 7.5 [52.7]	0.5037
Mental health	0–100	57.8 ± 3.9 [57.8]	49.1 ± 10.2 [51.3]	**0.0005**	50.4 ± 9.2 [53.5]	48.1 ± 11.2 [50.9]	0.5371
**Mental component summary**	0–100	57.2 ± 3.8 [57.7]	51.9 ± 9.6 [54.6]	**0.0188**	51.7 ± 10.4 [55.0]	52.1 ± 9.2 [54.6]	0.9181

**Short-form questionnaires, mean (SD)[§]**							
Morning stiffness	0–4	0.2 (0.3)	1.7 (0.8)	**<0.001**	1.3 (0.8)	2.0 (0.6)	**0.025**
Afternoon stiffness	0–4	0.1 (0.3)	1.4 (0.7)	**<0.001**	0.9 (0.6)	1.7 (0.5)	**0.0016**
Fatigue scale	0–10	0.6 (0.9)	4.3 (1.8)	**<0.001**	3.6 (1.4)	4.9 (2.0)	0.06
Pain scale	0–100	0.9 (1.6)	31.5 (18.1)	**<0.001**	21.5 (17.2)	40.1 (14.4)	**0.016**
PGA	0–100	n/a	33.2 (18.4)	**<0.001**	22.2 (17.1)	42.7 (13.9)	**0.0061**
**Time in bed, hours, mean (SD)**	N/A	8.3 (0.7)	8.0 (0.9)	0.11	8.1 (0.9)	7.9 (0.9)	0.34

Wilcoxon signed-rank test was used to compare the collection rates of matched RA vs control cohorts, and participants with severe vs moderate RA. *P*-values <0.05 were considered to be statistically significant and are marked in bold.

*Two participants with RA withdrew from the study before contributing data; therefore, to preserve matching groups for statistical tests, the matched controls were not included in these analyses; [†]RAPID-3 score ≤12; [‡]RAPID-3 score >12; [§]unless otherwise specified, a higher score corresponds to decreased QoL; **a higher score corresponds to increased QoL and a score of <30 indicates severe fatigue; [††]PROMIS pain and sleep scores are based on a normal distribution generated from national US data with a mean of 50 and an SD of 10, and a higher score indicates decreased QoL; [‡‡]SF-36 scores are based on a normal distribution generated from global data with a mean of 50 and an SD of 10, and a higher score indicates increased QoL.

FACIT, Functional Assessment of Chronic Illness Therapy—Fatigue; HAQ-DI, Health Assessment Questionnaire Disability Index; N/A, not available; PGA, patient global assessment; PROMIS, Patient-Reported Outcomes Measurement Information System; QoL, quality of life; RA, rheumatoid arthritis; RASIQ, RA Symptom and Impact Questionnaire; RAPID-3, Routine Assessment of Patient Index Data 3; SD, standard deviation; SF-36, Short Form 36.

Supplementary Table 3. Schedule of assessments for guided tests and PRO measures

Tests	Day 0	Day 1	Days 2–6	Day 7	Days 8–13	Day 14	Last visit
GENERAL QUESTIONS							
Demographics (6 questions)	2 min						
Clinical characteristics (7 questions)	2 min						
FLEXIBLE ASSESSMENTS, ANY TIME OF THE DAY							
RA Symptom & Impact Q (16 items)		4 min		4 min		4 min	
PROMIS Pain Interference (11 items)		3 min		3 min		3 min	
HAQ-DI (23 items)		6 min		6 min		6 min	
PROMIS Sleep Disturbance (7 items)		2 min		2 min		2 min	
FACIT-Fatigue (13 items)		3 min		3 min		3 min	
SF-36 (36 items)		9 min		9 min		9 min	
MORNING ASSESSMENTS							
Sleep and wake times (2 items)		1 min	1 min	1 min	1 min	1 min	
**Objective guided tests**							
9-hole peg test		1 min	1 min	1 min	1 min	1 min	
Wrist ROM test		1 min	1 min	1 min	1 min	1 min	
Gait test		1 min	1 min	1 min	1 min	1 min	
Lie-to-stand test		1 min	1 min	1 min	1 min	1 min	
Sit-to-stand test		1 min	1 min	1 min	1 min	1 min	
**Subjective assessments**							
Pain VAS (1 item)		<1 min	<1 min	<1 min	<1 min	<1 min	
Joint-pain map (variable)		1–5 min	1–5 min	1–5 min	1–5 min	1–5 min	
AFTERNOON ASSESSMENTS							
**Objective guided tests**							
9-hole peg test		1 min	1 min	1 min	1 min	1 min	
Wrist ROM test		1 min	1 min	1 min	1 min	1 min	
Gait test		1 min	1 min	1 min	1 min	1 min	
Lie-to-stand test		1 min	1 min	1 min	1 min	1 min	
Sit-to-stand test		1 min	1 min	1 min	1 min	1 min	
**Subjective assessments**							
Pain VAS (1 item)		<1 min	<1 min	<1 min	<1 min	<1 min	
Joint-pain map (JMAP)		1–5 min	1–5 min	1–5 min	1–5 min	1–5 min	
Morning stiffness severity: Q11 from RA Symptom & Impact Q (1 item)			<1 min		<1 min		
Morning stiffness duration: Q12 from RA Symptom & Impact Q (1 item)			<1 min		<1 min		

Afternoon stiffness severity: Q13 from RA Symptom & Impact Q (1 item)			<1 min		<1 min		
Fatigue question (1 item)		<1 min	<1 min	<1 min	<1 min	<1 min	
Patient global assessment VAS (1 item)		<1 min	<1 min	<1 min	<1 min	<1 min	
STUDY FEEDBACK QUESTIONS							
Questions on patient experience with the study (~30 questions)							8 min
**Approximate total time (min/day)**	**4**	**44–48**	**19–23**	**44–48**	**19–23**	**44–48**	**8**

Key:

Completed at the beginning or the end of the study only
Conducted once per week
Conducted once in the morning on any given day
Conducted twice on a given day, once in the morning, and once in the evening
Conducted once in the evening on any given day

For demographics, characteristics, and subjective assessments, the test length was calculated assuming ~1 minute to complete 4 items or questions (for the JMAP, it was assumed participants would fill the questionnaire for 5–20 joints at any given time). For each objective assessment, the test length calculation assumed ~1 minute per guided test. The questions on time of going to bed and getting out of bed were asked before all other questions on Days 1–14. The longer PRO measure questionnaires were made available for participants to answer at the beginning of Days 1, 7, and 14. Participants had flexibility to complete these questionnaires at any point and in any order during the day but were encouraged to complete them before the guided tests.

FACIT-Fatigue, Functional Assessment of Chronic Illness Therapy—Fatigue; HAQ-DI, Health Assessment Questionnaire-Disability Index; PRO, patient-reported outcome; PROMIS, Patient-Reported Outcomes Measurement Information System; Q, questionnaire; RA, rheumatoid arthritis; ROM, range of motion; SF-36, Short Form 36; VAS, visual analog scale.

Supplementary Table 4. Participant inclusion and exclusion criteria

Inclusion criteria	Exclusion criteria
– Clinically verified diagnosis of moderate to severe RA, with severity assessed using RAPID-3 (participants with RA only)<br><br>– At least 21 years of age<br><br>– Capable of giving signed informed consent<br><br>– Able and willing to perform the predefined guided tests at the start of the study (participants were not removed from the study if they were unable to perform the tests partway through)<br><br>– Able to follow audio instructions from an iPhone<br><br>– Sufficient level of English language to ensure ability to understand mobile app and questionnaires (judged by RMG PI and/or clinic staff)	– Prior or current diagnosis of a rheumatological disorder, inflammatory disorder, malignancy, or other relevant diseases (controls only)<br><br>– Current drug or alcohol abuse or dependence as recorded in patient charts<br><br>– Pregnant or lactating<br><br>– History of surgery within the past 6 months, unless fully recovered<br><br>– History of other inflammatory rheumatologic or systemic autoimmune disorder that may confound the study<br><br>– History of movement disorders, other neurological disorders, postural hypotension, unexplained syncope, or conditions that may impact the study<br><br>– Use of a wheelchair, walking aids, or artificial limbs, history of severe skin allergy, or have any active implantable device or pacemaker

PI, principal investigator; RAPID-3, Routine Assessment of Patient Index Data 3; RMG, Reliant Medical Group.

Supplementary Figure 1. Associations between dominant wrist angular velocity and dominant wrist pain (a), wrist ROM and stiffness (b), and sit-to-stand time and stiffness (c)

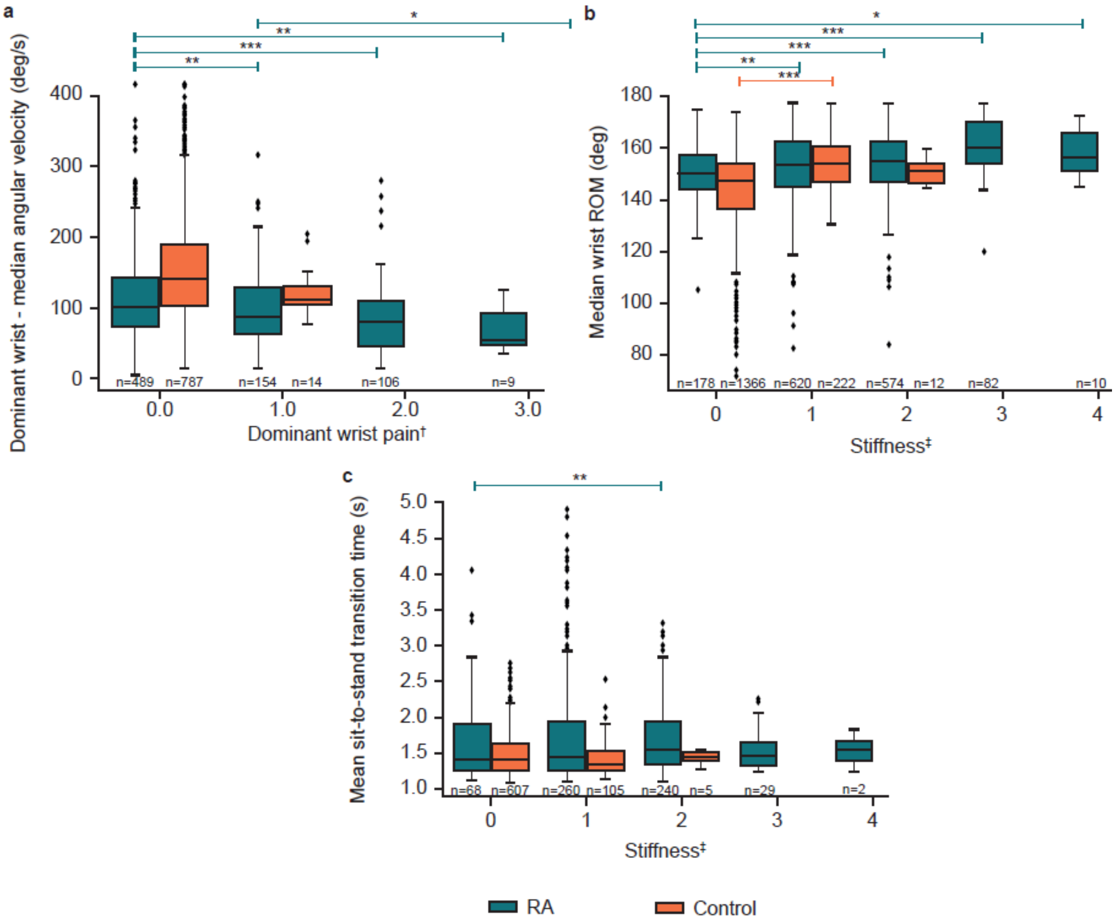

One-way ANOVA was used to compare differences in guided test performances and PRO measures. One-way ANOVA post hoc analyses were used to compare differences in guided tests between groups based on symptom severity as determined by PRO measures (**P*<0.05, ***P*<0.01, ****P*<0.001).
†Dominant wrist pain was determined by the JMAP score ranging from "no pain" (0) to "severe pain" (3); ‡Stiffness was assessed using a single-item question on the severity of morning stiffness experienced by the participant on a given day, with 5 response options provided, ranging from no stiffness (0) to very severe (4).
ANOVA, analysis of variance; deg, degrees; deg/s, degrees per second; JMAP, joint-pain map; PRO, patient-reported outcome; RA, rheumatoid arthritis; ROM, range of motion; s, seconds.

Supplementary Figure 2. Effect of time on lie-to-stand (a), wrist angular velocity (b), and sit-to-stand (c) guided tests

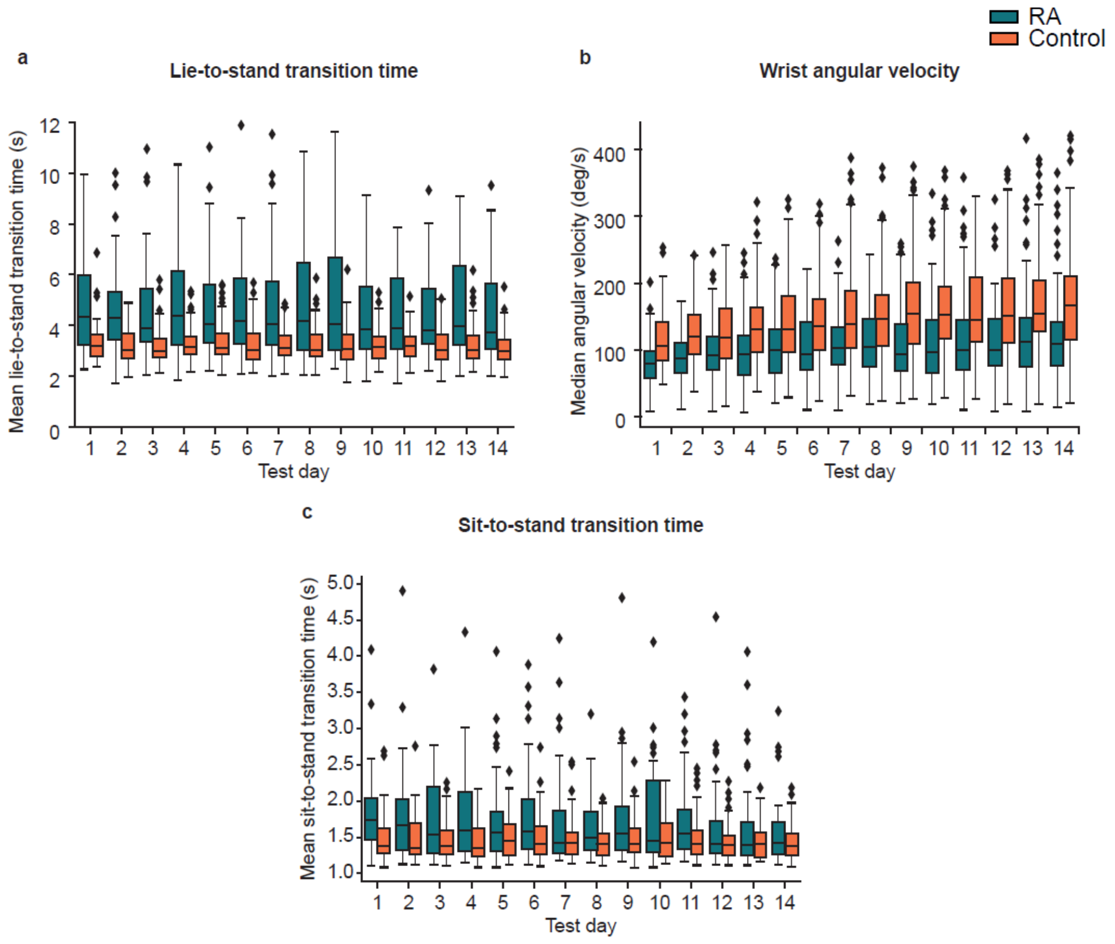

A univariate mixed effects linear model was used to assess association of time with wrist angular velocity, sit-to-stand, and lie-to-stand transition times.
Deg/s, degrees per second; RA, rheumatoid arthritis; s, seconds.

Supplementary Figure 3. Mobile application screenshots of the homepage (a), afternoon tasks (b), example survey questions (c), survey questions using pain scale (d), joint-pain map (JMAP) questions (e), and the lie-to-stand test (f)

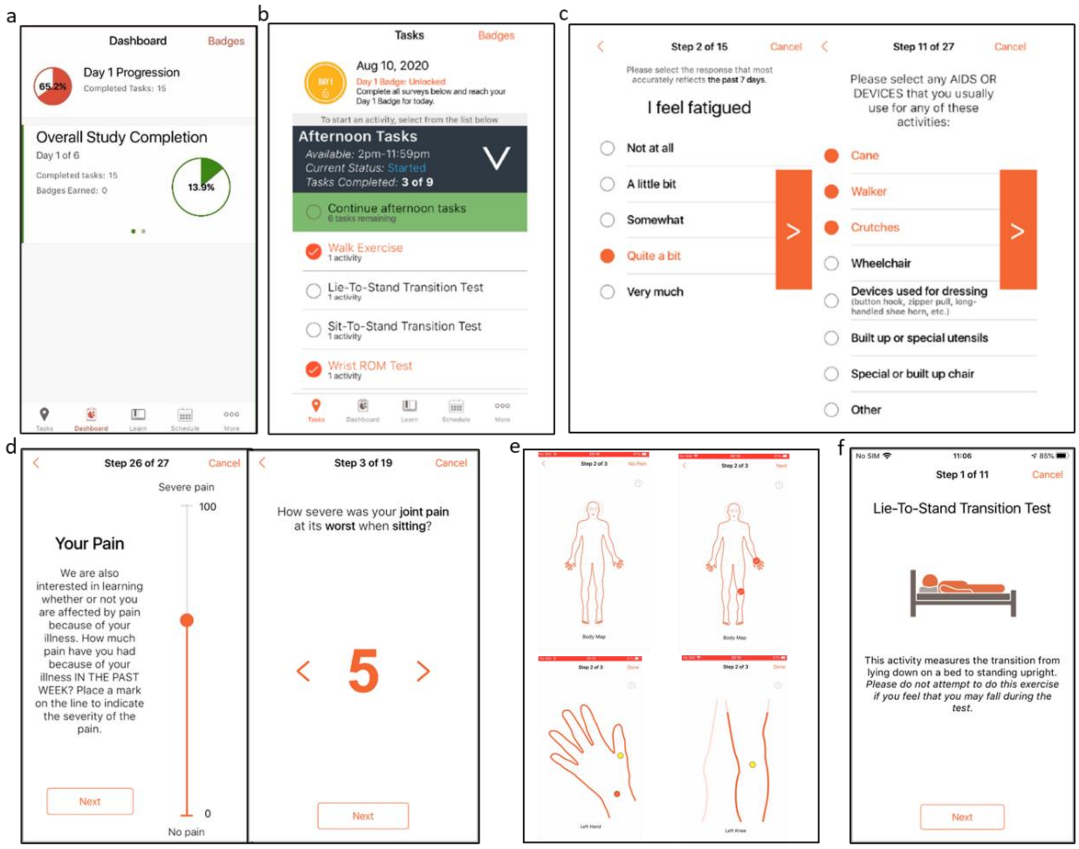

Supplementary Figure 4. Simplified algorithm flow chart of the lie-to-stand test

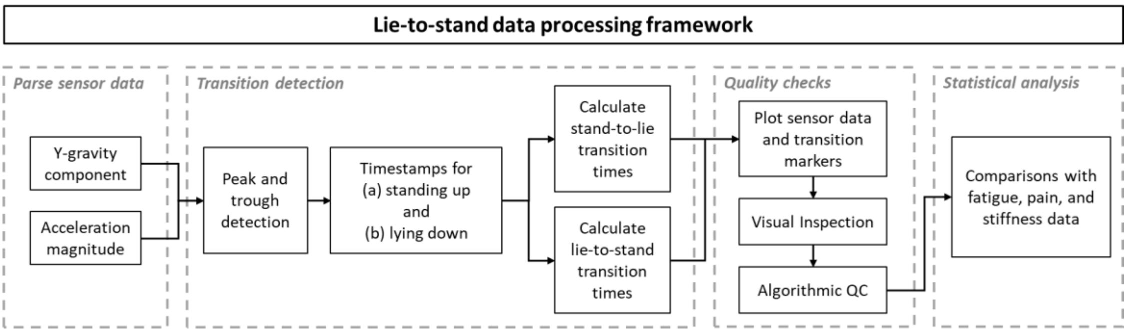

QC, quality control.
